# Visual response and anatomical changes on sequential spectral-domain optical coherence tomography in birdshot chorioretinopathy treated with local corticosteroid therapy

**DOI:** 10.1186/s40942-016-0034-y

**Published:** 2016-02-20

**Authors:** Marisa Gobuty, Mehreen Adhi, Sarah P. Read, Jay S. Duker

**Affiliations:** 1 New England Eye Center, Tufts University School of Medicine, 800 Washington Street, Boston, MA 02111 USA; 2 Department of Electrical Engineering, Massachusetts Institute of Technology, Cambridge, MA USA

**Keywords:** Birdshot chorioretinopathy, Spectral-domain optical coherence tomography, Retinal thinning, Cystoid macular edema, Choroidal thinning, Choroidal thickness, Choroidal vasculature, Corticosteroids, Immunosuppression

## Abstract

**Background:**

Birdshot chorioretinopathy is a chronic bilateral inflammatory disease of unknown etiology characterized by bilateral retinal vasculitis, mild to moderate vitritis, retinal vascular leakage, cystoid macular edema (CME), and typical “birdshot” chorioretinal lesions. Typically, patients with birdshot chorioretinopathy are treated with systemic immunosuppressive and/or corticosteroid therapy in an effort to minimize loss of vision. Spectral-domain OCT (SD-OCT) has shown regional or generalized photoreceptor loss in addition to both retinal as well as choroidal thinning in these patients. The present study describes anatomical changes of the retina and alterations in choroidal thickness and vasculature on sequential spectral-domain optical coherence tomography (SD-OCT) in 4 patients with birdshot chorioretinopathy treated with local corticosteroids.

**Methods:**

A retrospective observational case series identified 4 consecutive patients (8 eyes) at New England Eye Center, Boston diagnosed with birdshot chorioretinopathy according to the research criteria of the international consensus conference that were managed by a single retina specialist and treated exclusively with local corticosteroid therapy (intravitreal/sub-tenon injections) without systemic immunosuppression. All patients underwent longitudinal SD-OCT imaging with both the 512 × 128 cube scan and the 1-line raster protocol. A chart review was performed to review the visual response to treatment. Two independent observers analyzed sequential SD-OCT images for retinal parameters such as occurrence of CME at any time during the course of disease, presence of retinal thinning and presence of hyper-reflective foci within the retina, and choroidal parameters including its thickness and its vasculature.

**Results:**

Mean age of the patients at diagnosis was 47 years (26–60 years). Mean duration of follow-up was 96 months. All patients were HLA-A29 positive. Visual acuity remained stable in 75 % of eyes, 63 % eyes had central retinal thinning, 75 % eyes had hyper reflective foci within the retina and 75 % eyes had CME during follow-up. Mean total sub-foveal choroidal thickness of all 8 eyes at the time of the last SD-OCT was significantly lower than at initial SD-OCT (p = 0.03).

**Conclusions:**

This case series suggests that treatment with local corticosteroids may have good visual outcome despite retinal and choroidal thinning. Future longitudinal studies are necessary to further determine the benefits of local corticosteroid therapy.

## Background

Birdshot chorioretinopathy is a chronic bilateral inflammatory disease of unknown etiology that is strongly associated with HLA-A29 genotype [1]. It is characterized by bilateral retinal vasculitis, mild to moderate vitritis, retinal vascular leakage, cystoid macular edema (CME), and typical “birdshot” chorioretinal lesions. The latter are characterized as multiple cream-yellow colored hypo-pigmented choroidal infiltrates [1–4]. Overtime, vascular attenuation and atrophy of the retinal pigment epithelium (RPE), photoreceptors, choroid and optic nerve develop, that can significantly compromise vision [1–4].

A progressive loss of vision occurs in patients with birdshot chorioretinopathy over 2–3 decades [2, 4, 5]. Disease course is generally monitored with changes in visual acuity over time, cellular reaction within the anterior chamber and vitreous, leakage of the retinal vasculature and the appearance of the hallmark birdshot lesions. Visual acuity alone has not been shown to significantly correlate with clinical inflammation and therefore does not fully reflect disease severity in birdshot chorioretinopathy [6]. Fluorescein angiography and indocyanine green angiography have been utilized to visualize the vascular architecture in these patients and monitor the extent of vascular leakage as a proxy for disease activity [7, 8]. In addition, optical coherence tomography (OCT), visual fields and electroretinography (ERG) can also be used to monitor disease activity [9–12]. Using spectral-domain OCT (SD-OCT) regional or generalized photoreceptor loss in addition to both retinal as well as choroidal thinning has been observed [3]. A recent study showed that OCT is a valuable tool to quantify the retinal degeneration in birdshot chorioretinopathy patients [13]. More recently, optical coherence tomography angiography (OCTA) has demonstrated the absence of choriocapillaris beneath the disrupted RPE within the birdshot lesions and within the retinal vasculature telangiectatic vessels and dilated capillaries with increased intercapillary space were identified [14].

Typically, patients with birdshot chorioretinopathy are treated with systemic immunosuppressive and/or corticosteroid therapy in an effort to minimize loss of vision [5, 15–20]. Of late, intravitreal sustained release devices show improved vision and decreased inflammation in these cases [4, 21]. The present case series aims to study the visual outcome and anatomical changes such as the morphological features of the retina and alterations in choroidal thickness and vasculature on sequential SD-OCT over time in 4 patients with birdshot chorioretinopathy treated with local corticosteroid therapy.

## Methods

### Patients

A retrospective observational case series identified 4 consecutive patients (8 eyes) at the New England Eye Center, Tufts University School of Medicine, Boston diagnosed with birdshot chorioretinopathy according to the research criteria of the international consensus conference that were managed by a single retina specialist (JSD) and treated exclusively with local corticosteroid therapy (intravitreal/sub-tenon injections) without systemic immunosuppression [22]. All patients had bilateral disease and had at least 3 peripapillary birdshot lesions with anterior segment and/or vitreal inflammation. HLA typing for HLA-A29, fluorescein angiography and indocyanine green angiography for presence of vascular leakage, and OCT imaging to detect cystoid macular edema (CME) were performed to support the diagnosis. In all cases, visual fields were performed to follow the progression of disease. Ancillary testing ruled out other causes of multifocal choroidal lesions when indicated. Retrospective chart review identified the disease course and the visual response to the administered treatment. Sequential SD-OCT images were analyzed to determine the anatomical changes over time. This study received approval from the Institutional Review Board. The research adhered to the tenets of the Declaration of Helsinki and complied with the Health Insurance Portability and Accountability Act of 1996. Informed consent was considered exempt for this study due to its retrospective design by the Institutional Review Board.

### SD-OCT imaging and analysis of the retinal and choroidal changes

All patients underwent longitudinal SD-OCT imaging with both the 512 × 128 cube scan and the 1-line raster protocol on Cirrus HD-OCT (Carl Zeiss Meditec Inc, Dublin, California, USA). The 1-line raster protocol is a 6-mm line scan that acquires 20 frames at the same retinal location that are then averaged together to increase the signal-to-noise ratio. The enhanced depth imaging (EDI) protocol could not be employed, as it was not available on the Cirrus device at the time most of these scans were performed.

Two independent OCT raters (MA and SPR) experienced in analyzing SD-OCT images studied the morphology of the retina and choroid on sequential SD-OCT images in all patients [23]. The retinal parameters analyzed included (1) occurrence of CME at any time during the course of disease, (2) presence of retinal thinning and (3) presence of hyper-reflective foci within the retina. The subfoveal total choroidal thickness and the subfoveal large choroidal vessel layer thickness were measured using previously described methods in all eyes [24–27]. Medium choroidal vessel layer/choriocapillaris layer thickness was calculated by subtracting the subfoveal large choroidal vessel layer thickness from the subfoveal total choroidal thickness [24–27].

### Statistical analysis

All data was expressed as mean ± standard error of the mean (SEM). The differences in the total choroidal thickness and thickness of the individual choroidal vascular layers from an initial SD-OCT to the recent most SD-OCT image (final SD-OCT) were analyzed using a paired t-test. Pearson correlation was used to determine correlation of central retinal thickness with the subfoveal total choroidal thickness and the subfoveal large choroidal vessel layer thickness. A 95 % confidence interval and a 5 % level of significance were adopted; therefore, results with a p value less than or equal to 0.05 were considered significant.

## Results

### Description of cases

Mean age of the patients at diagnosis was 47 years (26–60 years). Mean duration of follow-up was 96 months. All patients were HLA-A29 positive.

**Fig. 1:**
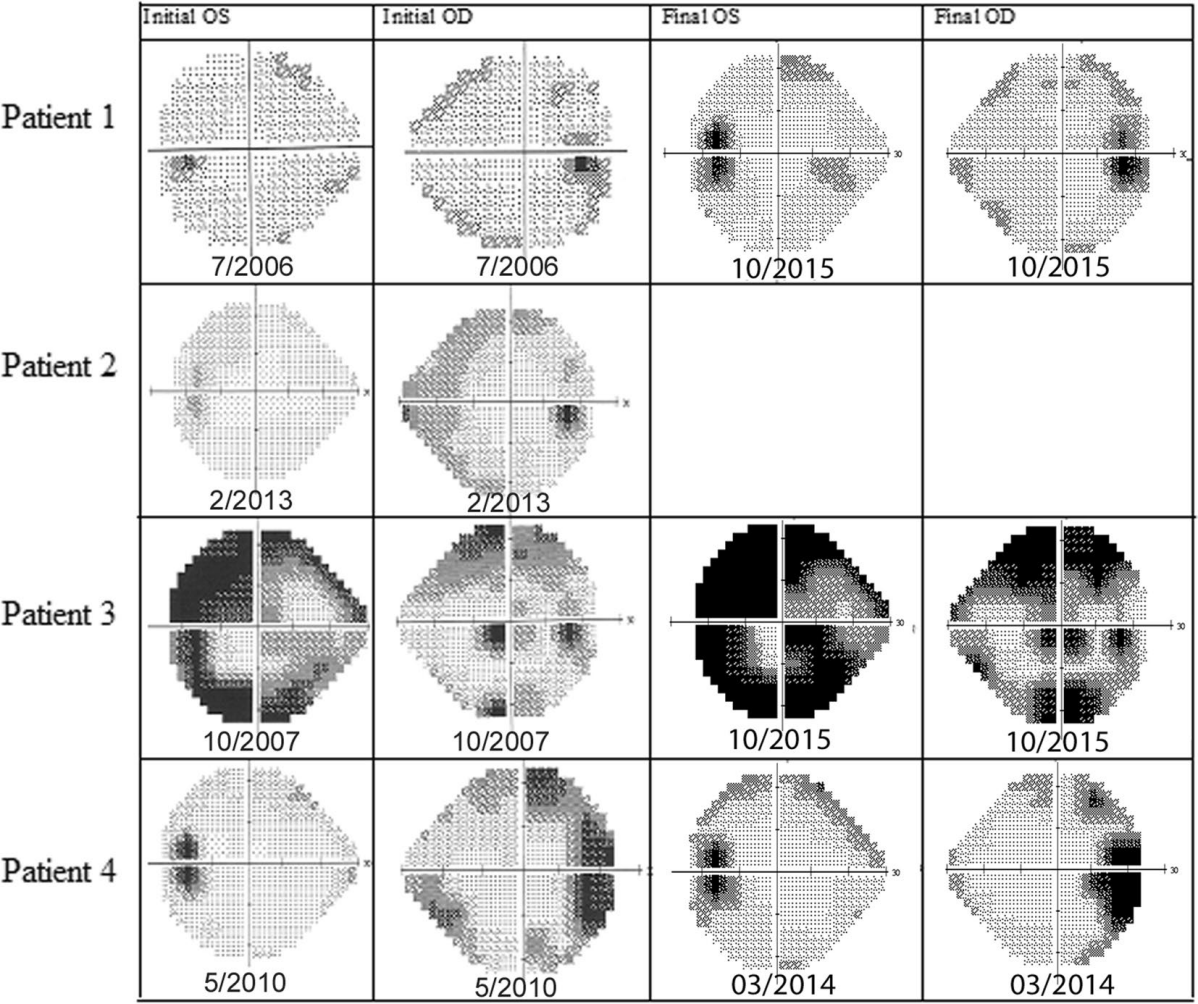
Initial and final visual field exams for all patients except patient 2 who only had an initial visual field exam. Patient 1 remained relatively stable. Patient 3 had progression showing dense arcuate scotomas bilaterally. Patient 4 showed mild improvement in the right eye

Patient 1 was diagnosed with birdshot chorioretinopathy at age 54. At initial presentation, his best-corrected visual acuity (BCVA) was 20/50 in the right and 20/30 in the left eye. OCT imaging showed no evidence of CME. Three years after presentation, he developed an extrafoveal choroidal neovascularization (CNV) in the left eye that was treated with focal laser therapy. He later developed CME bilaterally that was treated with bilateral intravitreal corticosteroid (triamcinolone 4 mg) injections every 6 months. A retinal detachment developed in his right eye that was treated with pneumatic retinopexy. At his recent follow-up in 2015, 11 years after his initial presentation, the patient had received 11 and 9 triamcinolone injections in his right and left eye respectively. He remained a glaucoma suspect in both eyes and is treated with dorzolamide-timolol. He underwent cataract surgery in 2009 and 2014. His BCVA was 20/50 in the right and 20/30 in the left eye, SD-OCT imaging showed retinal thinning without CME and visual fields remained relatively stable (Fig. 1).

**Fig. 2:**
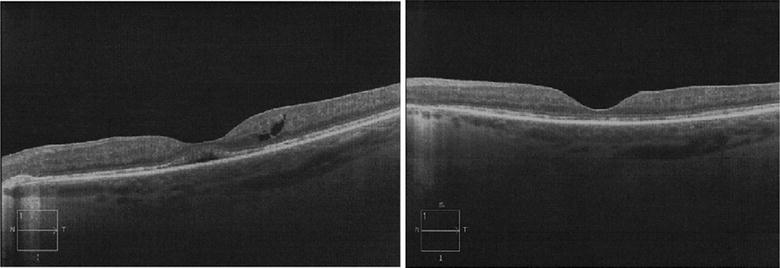
Sequential SD-OCT images of the right eye from patient 2 showing response to treatment with intravitreal triamcinolone acetate. Complete resolution of CME following an intravitreal triamcinolone acetate injection is seen in this patient. *SD-OCT* spectral-domain optical coherence tomography, *CME* cystoid macular edema, *mo* months, *IVK* intravitreal triamcinolone acetate

Patient 2 was diagnosed with birdshot chorioretinopathy at age 48. On presentation, BCVA was 20/30 in the right and 20/20 in the left eye. OCT showed CME in the right eye that was treated with a single subtenon triamcinolone injection (Fig. 2). The CME resolved with treatment, however, he developed secondary elevated intraocular pressure. At his recent follow up in 2013, his intraocular pressure was well controlled (11 bilaterally) on dorzolamide-timolol twice a day. BCVA in the right eye was 20/50 and that in the left eye was 20/30 and OCT showed no CME bilaterally.

Patient 3 (sister of patient 2, see above) was diagnosed with birdshot chorioretinopathy at another institution in 1993 at age 34 where she was treated with cyclosporine 300 mg daily. She first presented to the New England Eye Center in 1999 for a second opinion. At that time, she had been off immunosuppressive medications for 2 years and her BCVA was 20/70 in the right and 20/60 in the left eye. OCT showed prominent CME and cystic changes bilaterally. Treatment was deferred 3 years for pregnancy. Three years later, she was treated with intravitreal triamcinolone injections bilaterally. She received a total of four injections in her right eye and five injections in her left eye for recurring CME and inflammation over the next 13 years. She developed intraocular hypertension that has been managed with dorzolamide-timolol. She underwent cataract surgery in the right eye in 2010 and in the left eye in 2003. At her recent follow-up in 2015, the BCVA was 20/40 in the right and 20/200 in the left eye and OCT showed no evidence of CME. Her VF showed dense arcuate scotomas bilaterally.

Patient 4 was diagnosed with birdshot chorioretinopathy 28 years ago at another institution. She first presented to the New England Eye Center at age 54 for a second opinion. At presentation, her BCVA was 20/40 in the right and 20/200 in the left eye. OCT imaging showed minimal CME in the left eye treated with intravitreal triamcinolone injection. She has received two intravitreal triamcinolone injection in the left and one in her right eye. She underwent cataract surgery in her right eye in 2013 and in her left eye in 2010. At her recent follow up in 2015, her visual fields remained stable with mild improvement in the right eye and on OCT thinning was noted bilaterally. The BCVA was 20/40 in the right and 20/70 in the left eye and OCT showed no evidence of CME.

### SD-OCT analysis of the retinal and choroidal changes

**Fig. 3:**
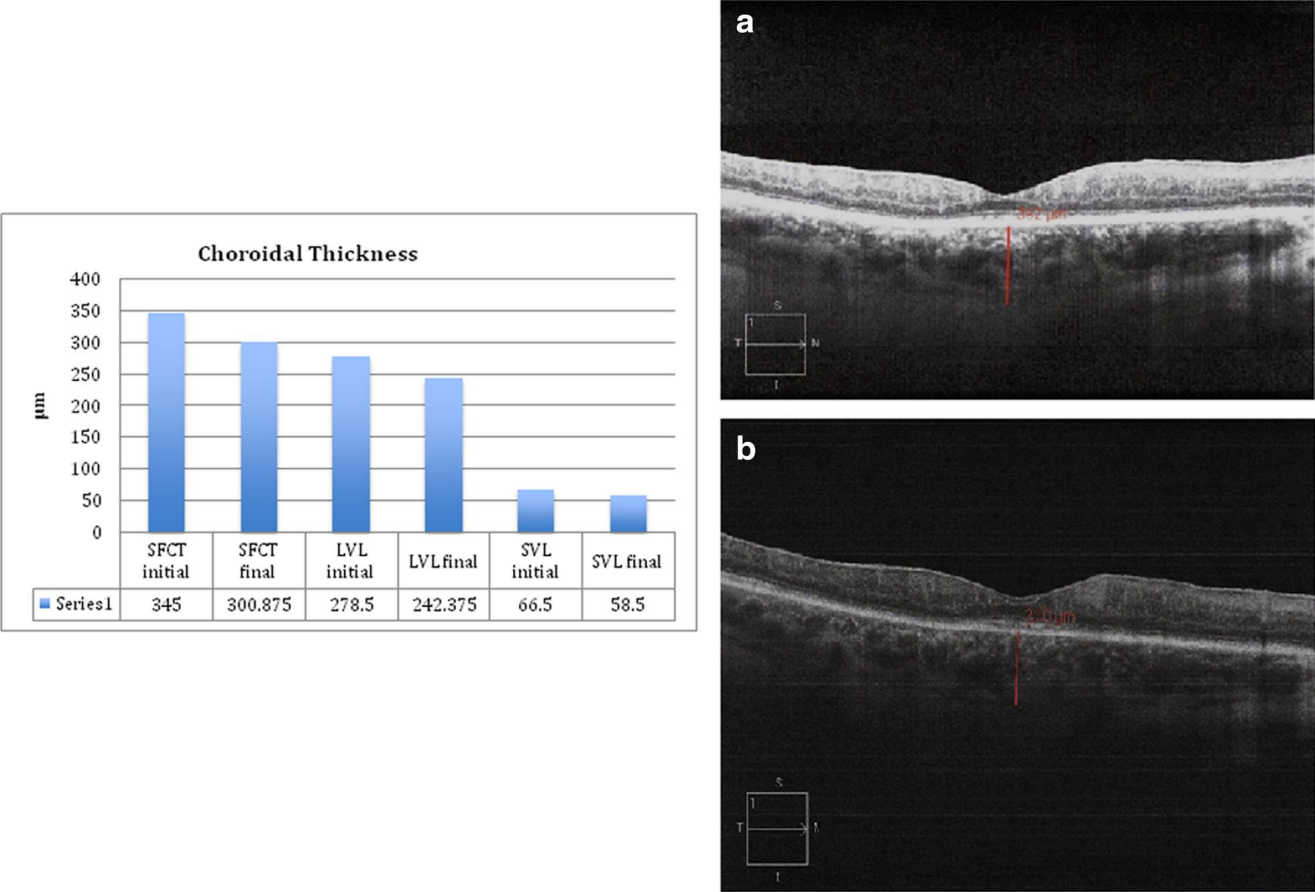
Analysis of the subfoveal total choroidal thickness and the thickness of the individual choroidal vascular layers. **a** Graph showing reduction in the subfoveal total choroidal thickness (SFCT), subfoveal large choroidal vessel layer (LVL) thickness and subfoveal medium choroidal vessel/choriocapillaris layer (SVL) thickness during follow-up. The *numbers* represent the mean thickness values. **b** SD-OCT images from patient 1 showing a reduction in the subfoveal total choroidal thickness over a 33-month period. *SD-OCT* spectral-domain optical coherence tomography, *mo* months

The morphological features of the retina analyzed on SD-OCT are described in Table 1. The demographic characteristics, treatment, and visual outcomes of patients are presented in Table 2. Choroidal thickness and vasculature changes at initial and final SD-OCT are charted in Fig. 3. The mean total sub-foveal choroidal thickness of the 8 eyes at the time of the last SD-OCT was significantly lower than at the initial SD-OCT (p = 0.03). There was no correlation of the mean central retinal thickness with the sub-foveal total choroidal thickness and the sub-foveal large choroidal vessel layer thickness (p = 0.83 and p = 0.10 respectively).

**Table 1:** Morphological features of the retina analyzed using spectral-domain optical coherence tomography (SD-OCT)

	Central retinal thinning	Hyper reflective foci within the retina	Cystoid macular edema (CME) anytime during disease course
Patient 1	1/2	2/2	2/2
Patient 2	0/2	0/2	1/2
Patient 3	2/2	2/2	2/2
Patient 4	2/2	2/2	1/2
Total %	62.5	75	75

**Table 2:** Demographic characteristics, treatment, and visual outcomes of patients with birdshot chorioretinopathy

Patient	Onset presentation	Prior treatment	VA initial	VA final	Disease course OD	Disease course OS	Family history	HLA A29	Treatment
1	54 yo	None	20/50 OD	20/50 OD	VC, ERM, RD, CME, PCL	VC, ERM, CME, PCL, CNV^b^	No	Positive	Focal laser OS, pneumatic retinopexy OD, IVK × 711 OD, IVK × 9 OS
	54 yo		20/30 OS	20/30 OS					
2	48 yo	None	20/30 OD	20/50 OD	ERM, CME, PCL, glaucoma^c^	ERM, PCL	Yes	Positive	Subtenon triamcinolone acetate OD, DC secondary to glaucoma
	48 yo		20/20 OS	20/30 OS					
3	34 yo	Systemic immuno-suppressants^a^	20/70 OD	20/40 OD	ERM, CME, PCL	ERM, CME, PCL	Yes	Positive	IVK × 4 OD, IVK × 5 OS
	40 yo		20/60 OS	20/200 OS					
4	26 yo	None	20/40 OD	20/40 OD	VC, PVD, PCL	VC, PVD, CME, PCL	No	Positive	IVK × 1 OD, IVK × 2 OS
	54 yo		20/200 OS	20/70 OS					

*PCL* pale choroidal lesions, *ERM* epiretinal membrane, *RD* retinal detachement, *CME* cystoid macular edema, *CNV* choroidal neovascularization, *VC* vitreous cell, *C/D* cup to disk, *IVK* intravitreal triamcinolone acetate, *PRP* pan-retinal photocoagulation, *N/A* not applicable, *PVD* posterior vitreous detachment

^a^Cyclosporine, ^b ^extrafoveal, ^c ^non-compliant to glaucoma medication

## Discussion

The present case series describes the visual response in consecutive patients with birdshot chorioretinopathy treated with local corticosteroid therapy. It further describes the retinal morphology and alterations in total choroidal thickness and thickness of the individual choroidal vascular layers on sequential SD-OCT in these patients.

Traditionally, patients with birdshot chorioretinopathy are treated with systemic immunosuppressive medications and/or systemic corticosteroids [12, 15–20]. Regional or systemic corticosteroids combined with cyclosporine or replaced from the outset by immunosuppressive therapy reduce vascular leakage and improve vision in these patients [15–20]. The use of immunomodulatory treatment (IMT) is supported by recent evidence suggesting that patients in remission without IMT may have subclinical inflammation [28]. Since birdshot chorioretinopathy has not been associated with systemic manifestations, intraocular drug delivery is an alternative therapeutic option. A study showed that implantation of a fluocinolone acetonide-containing intraocular device in patients with birdshot chorioretinopathy improves vision and reduces inflammation [4]. While eliminating the need for systemic immunosuppressive therapy, this study showed an increase in incidence of corticosteroid-induced glaucoma with 100 % of patients experiencing intraocular pressure greater than 25 mmHg and all 19 phakic eyes eventually requiring cataract surgery [4]. The present study suggests that local corticosteroid therapy alone administered as needed for CME or signs of intraocular inflammation may be a useful treatment option for the management of birdshot chorioretinopathy. While the local side effects such as glaucoma and cataract formation are expected in some eyes owing to the need of multiple injections, the systemic side effect profile is minimal. In this study, the visual acuity remained stable in 6 of 8 (75 %) eyes and visual fields (Fig. 1) displayed relative stability with mild progression in 2 of 6 eyes (33 %) over a mean follow up period of 96 months. Six of the 8 (75 %) eyes had evidence of ocular hypertension and 6 of 8 (75 %) developed cataracts during the course of follow-up.

This study showed central retinal thinning in 5 of 8 (62.5 %) eyes with birdshot chorioretinopathy. CME occurred in 6 of 8 (75 %) eyes during the course of follow-up. Hyper-reflective spots within the retina were observed in 6 of 8 (75 %) eyes and may suggest retinal pigment migration and/or clumping of the photoreceptors over a region of the RPE and/or photoreceptor disruption [3, 30]. Interestingly, a thinning of the subfoveal total choroid and the individual choroidal vascular layers over time was observed on sequential SD-OCT images. Keane et al described a thinning of the medium choroidal vessels in some patients with birdshot chorioretinopathy when compared to normal subjects [3]. The present study suggests that the choroid thins out over time in birdshot chorioretinopathy and this thinning primarily involves the large choroidal vessel layer (Fig. 3). Furthermore, there was no correlation of central retinal thickness and subfoveal total choroidal thickness and the sub-foveal large choroidal vessel layer thickness in all patients. This is consistent with the belief that choroidal and retinal inflammation may occur independently in patients with birdshot chorioretinopathy [3]. A limitation of this study was the inability to perform enhanced depth imaging [29] scans but all eyes had a clearly visible choroidoscleral interface at the subfoveal location without EDI and good inter observer correlation of choroidal thickness measurements.

## Conclusions

In conclusion, the present case series describes the visual outcome and anatomical changes in 4 patients with birdshot chorioretinopathy managed by a single retina specialist (JSD) in a single setting using local corticosteroid therapy. It shows that local corticosteroid therapy is a valuable therapeutic option for treatment of birdshot chorioretinopathy and can eliminate and/or delay the need for systemic immunosuppression. While local corticosteroid therapy is helpful at preserving visual function, it does not appear to prevent the retinal and choroidal changes that presumably occur secondary to the disease progression. Future longitudinal studies looking at a larger number of patients followed over a long time are expected to further determine the benefits of local corticosteroid therapy in this disease.

## Abbreviations

SD-OCT: spectral-domain optical coherence tomography
OCT: optical coherence tomography
CME: cystoid macular edema
RPE: retinal pigment epithelium
BCVA: best-corrected visual acuity
CNV: choroidal neovascularization
EDI: enhanced depth imaging
